# Combined Percutaneous and Transurethral Lithotripsy for Forgotten Ureteral Stents With Giant Encrustation

**DOI:** 10.5812/numonthly.4087

**Published:** 2012-09-24

**Authors:** Seyed Mohammadreza Rabani

**Affiliations:** 1 Beheshti Teaching Hospital, Yasuj University of Medical Sciences, Yasuj, IR Iran

**Keywords:** Ureter, Stents, Encrustation, Nephrostomy, Percutaneous

## Abstract

**Background:**

Ureteral stents are widely used in many urologic practices. However, stents can cause significant complications including migration, fragmentation, and encrustation and it may possibly be forgotten. Successful management of a retained, encrusted stent requires combined endourological approaches.

**Objectives:**

To present our experience with the approaches for treating forgotten ureteral stents associated with giant stone formation.

**Patients and Methods:**

Seventy four patients with forgotten ureteral stents were managed by different open (nephrolithotomy and/or cystolithotomy), or endoscopic procedures in our center. Among these, 11 patients had severe encrustation (stones larger than 35 mm within the bladder or kidney) and seven patients of this group, presented at our department between July 2007 and December 2010. Combined endourological procedures percutaneous nephrolithotripsy (PCNL), cystolithotripsy (CLT), transurethral lithotripsy (TUL) were performed in one or 2 separate sessions. In these 7 patients the whole of the stents, especially both ends were encrusted. Initially, cystolithotripsy, retrograde ureteroscopy and TUL were performed in the dorsal lithotomy position. Following this, a gentle attempt was made to retrieve the stent with the help of an ureteroscopic grasper. In some cases the stent was grasped by a hemostat clamp out of the urethral meatus with a gentle traction to facilitate lithotripsy in the ureter and even in the kidney. Finally, a ureteric catheter was placed adjacent to the stent for injection of radio-contrast material to delineate the renal pelvis and the calyces. Then in the same session or later in another session the patient was placed in the prone position and PCNL of the upper coil of the encrusted stent along with calculus was done and the stent was removed.

**Results:**

In 5 out of seven patients, the initial indication for stent placement was for urinary stone disease after open nephrolithotomy and pyeloplasty in other centers and in two patients after TUL. All patients underwent the procedure (s) under spinal anesthesia and all received antibiotics in preoperative period. The only available source of energy in our center was pneumatic lithotripsy.

**Conclusions:**

Multiple endourological approaches or even open surgery are needed because of encrustations and the associated stone burden that may involve bladder, ureter and kidney. This may require single or multiple endourological sessions or rarely open surgical removal of the encrusted stents. Although, endourological management of these stents achieves success in majority of the cases with minimal complications, the best treatment that remains is prevention of this complication and to achieve this important point designing a recall system is suggested.

## 1. Background

Multiple ureteral stent shapes, sizes, compositions, and designs have been studied to decrease patient discomfort during ureteral stent placement and indwelling time. However, the precise etiology of pain during ureteral stent indwelling remains elusive (1). Encrustation is a well-established complication of retained biomaterials in the urinary tract. Severe stent encrustation is a potentially serious complication of prolonged indwelling ureteral stenting often managed by open surgery when endoscopic techniques are unsuccessful (2). Ureteral stents are widely used in urologic daily practices and they have proved to be an invaluable tool in the armamentarium of the urological surgeon, indicated in selected cases of transureteral lithotripsy (TUL), after surgical ureteral repairs (like pyeloplasty and uretero-ureterostomy), and also as an adjunct to ureteral reconstruction in renal transplantation that may help to reduce early postoperative complications such as leakage and stricture (3). Extrinsic ureteral obstruction can be managed successfully by ureteral stent placement. In many gynecological operations, ureteral stents may be used, too (4, 5). However, stents can cause significant complications including migration, fragmentation, and encrustation, and it may possibly be forgotten. Successful management of a retained, encrusted stent requires combined endourological approaches. Percutaneous nephrostolithotomy (PCNL), ureteroscopy (URS), and transurethral lithotripsy or percutaneous cystolithotripsy (PCCL), are often necessary for treating a severely encrusted stent and associated stone burden (6).

## 2. Objectives

Herein we report our experiences in endoscopic management of forgotten ureteral stents with large burden stone encrustation, excluding those with minimal encrustation or those patients that were manageable by simple endourologic techniques such as TUL and SWL.

## 3. Patiens and Methods

During the last 10 years, 74 patients with forgotten, encrusted ureteral stents were managed by different open (nephrolithotomy and/or cystolithotomy), or endoscopic procedures in our center. Among them, 11 patients (5 women and 6 men) had severe encrustation (stones larger than 35 mm within the bladder or kidney) and 7 patients of these group (including 4 females and 3 males) were presented in our department between July 2007 and December 2010 a period that proper endourologic instruments were available for us. The mean patient age was 44.6 years (range 32-56 years) and average indwelling time of the stent was 4.4 years (range 1-8 years). Poor compliance of patients was the reason for retention of these stents. Combined endourological procedures including PCNL, Cystolithotripsy (CLT), and retrograde ureteroscopy with intracorporeal lithotripsy were performed in one or 2 separate sessions. Retrograde ureteroscopy was performed using 8-9.8F semi rigid Wolf ureteroscope. PCNL was carried-out using a rigid 24F nephroscope. In these 7 patients, the stents in whole, especially both ends were encrusted. Initially, cystolithotripsy, retrograde ureteroscopy, and TUL were performed in dorsal lithotomy position. Then, a gentle attempt was made to retrieve the stent with the help of an ureteroscopic grasper. In some cases the stent was grasped by a hemostat clamp out of urethral meatus with a gentle traction to facilitate lithotripsy in ureter and even in kidney. Finally, a ureteric catheter was placed adjacent to the stent for injection radio-contrast material to delineate the renal pelvis and calyces. Then in the same session or another later session the patient was placed in prone position and PCNL of upper coil of encrusted stent along with calculus was applied and the stent was removed.

## 4. Results

In 5 out of 7 patients, the initial indication for stent placement was urinary stone disease after open nephrolithotomy and pyeloplasty in other centers, and in 2 patients after TUL. Ultrasonography, intravenous urography, or spiral CT scan were performed to determine inclusion criteria (at least 35 mm stone size). All patients underwent the procedure (s) under spinal anesthesia and all received antibiotics in preoperative period. Bladder part of the stones were fragmented transurethrally (in 4 patients) or by PCCL (in 3 patients). PCNL was carried out in all patients both for removal of the stones and the stents. All the stents were removed intact and from the percutaneous tract. No intra operative complication occurred in any patient and all patients remained stone free after these combined endourologic procedures. The only available source of energy in our center was pneumatic lithotripsy.

## 5. Discussion

Forgotten ureteral stents are observed in urologic practice because of poor compliance of the patient or failure of the physician to adequately counsel the patient. Retained ureteral stents with encrustation is a challenging problem for urologists. Imaging and assessment of the degree of stone burden is important before making any attempt to remove these stents. Very often, multiple endourological approaches or even open surgery are needed because of encrustations and associated stone burden that may involve bladder, ureter, and kidney. This may require single or multiple endourological sessions or rarely open surgical removal of the encrusted stents.

Any catheter material placed in the urinary tract provides a surface for bacterial colonization and, therefore, it is susceptible to encrustation with crystalline bacterial biofilm. So many efforts have been done to prevent and manage the complications of ureteral stents. I have accidentally found a catheter completely free of encrustation in the renal pelvis of a 46 years old lady during a ureterorenoscopic procedure to follow an upward migrated proximal ureteral stone. This catheter that was a simple feeding tube has been inserted 22 years before removal, (when she underwent open nephrolithotomy), but it is not a rule. Some investigators have offered a new strategy to improve the surface properties of ureteral stents. This novel surface effectively decreases friction, encrustation tendencies, and biofilm formation to prevent stent encrustation (7); others have suggested an electronic stent extraction reminder facility recall base to prevent forgotten stents (8).

Successful management of a forgotten encrusted ureteral stent requires combined endourological approaches. PCNL and URS are often necessary for treating a severely encrusted stent and associated stone burden. In this study we observed that the passively dilated ureters caused easy URS beside the indwelling catheter and facilitated stone access even within the renal pelvis and some calices. Gentle traction on the catheter by a clamp after passage of the catheter from the urethral meatus causes no danger of upper tract traumatization because this force is mostly focused on distal part of the stent and the elastic tendency of the stent precludes force transferring upward. Neglected stents still represent a challenge in urology: while endourology remains the best option for treatment, the management of ureteral stents should be based on follow-up and prevention, such as computerized warning and stent retrieval software system as examples (9).

Although, endourological management of these stents achieves success in majority of cases with minimal complications, the best treatment would be prevention from this complication and achieving this important goal, programming an effective recall system is suggested.
